# Phase Change Ge-Rich Ge–Sb–Te/Sb_2_Te_3_ Core-Shell Nanowires by Metal Organic Chemical Vapor Deposition

**DOI:** 10.3390/nano11123358

**Published:** 2021-12-10

**Authors:** Arun Kumar, Raimondo Cecchini, Claudia Wiemer, Valentina Mussi, Sara De Simone, Raffaella Calarco, Mario Scuderi, Giuseppe Nicotra, Massimo Longo

**Affiliations:** 1 CNR—Institute for Microelectronics and Microsystems, Via C. Olivetti 2, 20864 Agrate Brianza, Italy; arun.kumar@mdm.imm.cnr.it (A.K.); claudia.wiemer@mdm.imm.cnr.it (C.W.); 2 CNR—Institute for Microelectronics and Microsystems, Via Gobetti 101, 40129 Bologna, Italy; cecchini@bo.imm.cnr.it; 3 CNR—Institute for Microelectronics and Microsystems, Via del Fosso del Cavaliere 100, 00133 Rome, Italy; valentina.mussi@artov.imm.cnr.it (V.M.); sara.desimone@artov.imm.cnr.it (S.D.S.); raffaella.calarco@artov.imm.cnr.it (R.C.); 4 CNR—Institute for Microelectronics and Microsystems, Strada VIII 5, 95121 Catania, Italy; mario.scuderi@imm.cnr.it (M.S.); giuseppe.nicotra@imm.cnr.it (G.N.)

**Keywords:** MOCVD, VLS, phase-change memory, nanowires, core-shell, Ge–Sb–Te, Ge–Sb–Te/Sb_2_Te_3_

## Abstract

Ge-rich Ge–Sb–Te compounds are attractive materials for future phase change memories due to their greater crystallization temperature as it provides a wide range of applications. Herein, we report the self-assembled Ge-rich Ge–Sb–Te/Sb_2_Te_3_ core-shell nanowires grown by metal-organic chemical vapor deposition. The core Ge-rich Ge–Sb–Te nanowires were self-assembled through the vapor–liquid–solid mechanism, catalyzed by Au nanoparticles on Si (100) and SiO_2_/Si substrates; conformal overgrowth of the Sb_2_Te_3_ shell was subsequently performed at room temperature to realize the core-shell heterostructures. Both Ge-rich Ge–Sb–Te core and Ge-rich Ge–Sb–Te/Sb_2_Te_3_ core-shell nanowires were extensively characterized by means of scanning electron microscopy, high resolution transmission electron microscopy, X-ray diffraction, Raman microspectroscopy, and electron energy loss spectroscopy to analyze the surface morphology, crystalline structure, vibrational properties, and elemental composition.

## 1. Introduction

Chalcogenide materials have gained the interest of the microelectronic industry because of their fast data-storage speed, high endurance, reduced power consumption, high scalability, and cost effective production for the next generation of electronic phase change memories (PCM) [1,2,3]. The PCM data storage mechanism exploits the fast and reversible phase transitions of chalcogenide compounds, driven by electrical pulses, so that a resistivity contrast between the amorphous and crystalline phases is generated, to which digital information can be associated [4].

Over the last decades, several phase change compounds such as GeTe [5], Sb_2_Te_3_ [6,7], Ge_2_Sb_2_Te_5_ (GST) [8,9], and In–Sb–Te [10] have been successfully synthesized. Indeed, GST is a typical phase-change compound exhibiting fast phase transitions between the amorphous and crystalline states. Such a structural modification involves the switching between the low and high electrical resistivity states associated with the two phases of the compound [11,12].

In order to obtain better device performances, the properties of the phase change materials can be optimized. GST exhibits a crystallization temperature (T_c_) of about 150 °C with an activation energy of 2.5 eV [13], which is considered not enough to guarantee suitable data retention performances, especially for automotive applications. However, it has been found that the T_c_ can be varied by modifying the chemical composition of GST [14,15] or by introducing proper dopants such as carbon [16], nitrogen [17], titanium [18], and arsenic [19]. A large increase in T_c_ can be obtained by introducing a higher Ge content than that of GST [14]. This modification strongly improves the thermal stability of the PCM, leading to high data retention. Compared to GST and GeTe, Sb_2_Te_3_ exhibits a lower T_c_, but faster reversible switching. Employing phase change alloys with different transition properties for the realization of memory cells based on heterostructures can improve the overall PCM performances as well as allow key studies on the role played by the heterointerface in the resistive switching mechanism. In fact, multilevel PCM cells based on Ge_2_Sb_2_Te_5_/Sb_2_Te_3_ and GeTe/Sb_2_Te_3_ have been reported for the multilayer geometry [20,21]. An intrinsic advantage of cell down scaling is obtained by reducing the programming volume, hence lowering the energy needed for the phase transitions, therefore the power consumption. In this regard, nanowires (NWs) offer promising perspectives due to their low dimensionality and single crystalline structure [12,22,23,24,25,26,27,28,29,30]. However, up to now, the synthesis and the physical–chemical characterization of Ge(Sb)Te/Sb_2_Te_3_ core-shell NWs have not been reported, except for our recent study on GeTe/Sb_2_Te_3_ core shell NWs [31].

For this reason, we focused on the synthesis of a Ge-rich Ge–Sb–Te/Sb_2_Te_3_ NW based heterostructure, which could meet the industrial specifications from the point of view of the thermal stability of PCMs. The implementation of such a Ge-rich Ge–Sb–Te/Sb_2_Te_3_ heterostructure in final devices is still demanding, since it involves not only a precise NW positioning and contacting, but also a high control of the Ge–Sb–Te composition during the manufacturing process. Nevertheless, such a bottom-up route can avoid the most critical stage in the fabrication of the device, namely the pattering of the cells [32,33]. Moreover, the core-shell NWs are expected to be a useful term of comparison with the equivalent PCM cells formed by planar heterostructures, offering a direct device access with the additional benefit of a relatively simple PCM cell that can be obtained by metal contact definition on the NWs [13,15].

In this work, we report on the bottom-up, Au catalyzed, metal-organic chemical vapor deposition (MOCVD) growth of Ge-rich Ge–Sb–Te/Sb_2_Te_3_ core-shell NWs. An extensive analytical characterization of the obtained core-shell heterostructures was performed. The core-shell NWs were synthesized by self-assembling the Ge-rich Ge–Sb–Te core, which was subsequently coated with the Sb_2_Te_3_ shell, conformally overgrown. The growth study led to optimized core-shell NWs and the assessment of their morphological, compositional, and structural properties, in light of their functional analysis.

## 2. Experimental Methods

The NW growth was performed by an Aixtron AIX200/4 MOCVD tool (Aixtron SE, Herzogenrath, Germany), employing the vapor–liquid–solid (VLS) mechanism catalyzed by different Au nanoparticles (NPs) with average sizes of 10 nm, 20 nm, 30 nm, and 50 nm. The Au NPs, in a colloidal solution from Ted Pella© (Redding, CA, USA), were dispersed on Si (100) and SiO_2_/Si substrates via a simple drop-casting method. The native oxide on the Si (100) substrates was removed by immersion in a HF 5% solution prior to dispersion of the Au NPs. The employed metal-organic sources were of electronic-grade tetrakisdimethylamino germanium (Ge[N(CH_3_)_2_]_4_, TDMAGe), antimonytrichloride (SbCl_3_), diisopropyltelluride ((C_3_H_7_)_2_Te, DiPTe), and bis(trimethylsilyl) telluride (Te(SiMe_3_)_2_, DSMTe), carried to the MOCVD reactor by an ultra-pure N_2_ process gas. The Ge-rich Ge–Sb–Te core NWs were obtained by employing the TDMAGe, SbCl_3_, and DiPTe precursors with the reactor pressure (P), reactor temperature (T), and growth duration (t) ranging in the values of 50–300 mbar, 300–400 °C, and 60–120 min, respectively. The Sb_2_Te_3_ shell overgrowth on the Ge-rich Ge–Sb–Te core NWs was performed at room temperature, P = 15 mbar, t = 90 min, SbCl_3_ partial pressure = 2.23 × 10^−4^ mbar, and DSMTe partial pressure = 3.25 × 10^−4^ mbar.

The surface morphology of the obtained NWs was investigated by a ZeissR©Supra40 field-emission scanning electron microscope (FE-SEM) (Cark Zeiss, Oberkochen, Germany) in plan as well as in cross-section mode. X-ray diffraction (XRD) experiments with an ItalStructures HRD3000 diffractometer system (Ital Structures Sas, Riva de Garda, Italy) were performed to pattern the average crystal structure of the obtained NWs. The obtained XRD patterns were analyzed using a best-fit process based on the Rietveld method.

High resolution transmission electron microscopy (HR-TEM) was employed to investigate the local microstructure, elemental composition, and the growth direction. For the TEM characterization, the as-synthesized NWs from the substrate were transferred directly on the TEM grid by mechanical rubbing. TEM characterization was carried out using a Cs-probe-corrected TEM JEOL ARM200CF microscope (JEOL Ltd., Akishima, Japan), operated at 200 keV and equipped with a GIF Quantum ER energy filter from GATAN (Gatan Inc., Pleasanton, CA, USA) for electron energy loss spectroscopy (EELS). Micro Raman characterizations were carried out by means of a Thermo Scientific DXR2xi Raman Imaging Microscope (Thermo Fischer Scientific, Waltham, MA, USA), coupled with a 532 nm excitation laser and a 50× objective. The obtained spectra were recorded after 40 accumulations of 20 ms acquisitions.

## 3. Results and Discussion

Initially, we investigated the growth of the Ge-rich Ge–Sb–Te core NWs. In this regard, we systematically explored the deposition on both Si (100) and SiO_2_/Si substrates with different Au NP sizes and it has to be noted that, depending on the precursor choice and deposition condition, different NW morphology, density, and compositions could be obtained from pure Ge to Sb_2_Te_3_ NWs (see Supplementary Materials, Figures S1–S6). Table S1, summarizes the growth parameters related to SEM images displayed in Figure S1, Supplementary Materials. In fact, at T = 400 °C, P = 50 mbar, and t = 60 min, the NWs with the best morphological characteristics in terms of length and density were achieved. Starting from this point, we obtained Ge-rich Ge–Sb–Te core NWs, with TDMAGe, SbCl_3_, and DiPTe bubblers’ partial pressures set to 3.35 × 10^−3^ mbar, 5.12 × 10^−5^ mbar, and 8.58 × 10^−3^ mbar, respectively. The plan view SEM images on Ge-rich Ge–Sb–Te core NWs grown with 10 nm, 20 nm, 30 nm, and 50 nm Au NPs on SiO_2_/Si, and Si (100) substrates demonstrated the formation of a good density of Ge-rich Ge–Sb–Te NWs via the VLS mechanism (see Supplementary Materials, Figure S5). The obtained NWs on SiO_2_/Si were found to have an average length and diameter distribution up to 1.40 µm and 100 nm, respectively (see Supplementary Materials, Figure S7).

**Figure 1:**
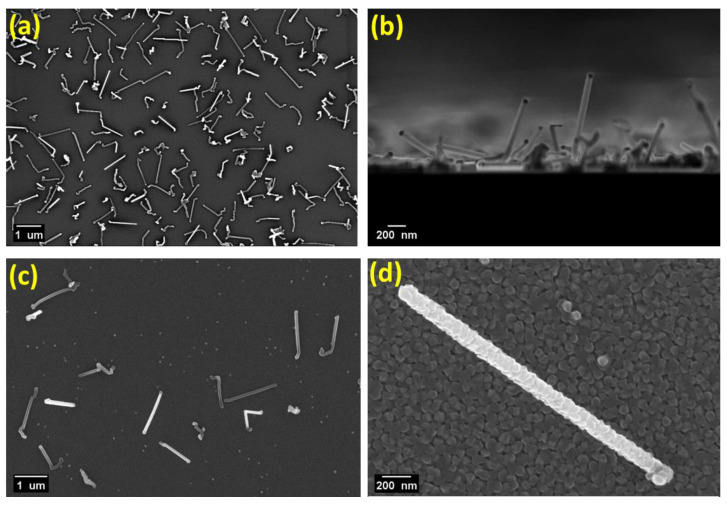
SEM images in (**a**) plan, (**b**) cross-section view of Ge-rich Ge–Sb–Te core NWs; and (**c**,**d**) plan view of Ge-rich Ge–Sb–Te/Sb_2_Te_3_ core-shell NWs on a SiO_2_/Si substrate, with different magnifications, respectively.

Figure 1a,b shows the planar and cross-sectional SEM images for Ge-rich Ge–Sb–Te core, and Ge-rich Ge–Sb–Te/Sb_2_Te_3_ core-shell NWs catalyzed by Au NPs of 50 nm size, respectively. Interestingly, the SiO_2_/Si surface exhibited a decent density of NWs all over the substrate (Figure 1a). The VLS growth mechanism in the obtained NWs could be confirmed by the presence of the catalyst Au NPs at the tips of the NWs.

In the second step of the core-shell fabrication, the Ge-rich Ge–Sb–Te NWs were coated with a Sb_2_Te_3_ deposition under the MOCVD growth conditions: T = room temperature, P = 15 mbar, t = 90 min, leading to the conformal growth of a uniform and continuous layer all over the core NWs, irrespectively of the different NP sizes (see Figure 1c,d and Supplementary Materials, Figure S6e–h). For the shell, it was necessary to lower the deposition temperature down to room temperature to obtain the conformal NW coating, although with a granular morphology. The obtained Ge-rich Ge–Sb–Te/Sb_2_Te_3_ core-shell NWs exhibited a conformal overgrowth of about 30 nm, with approximate inclusive diameters ranging from 90 nm to 130 nm. The obtained results showed that, with the appropriate growth condition selection, NWs with relatively high density and reproducibility can be achieved.

**Figure 2:**
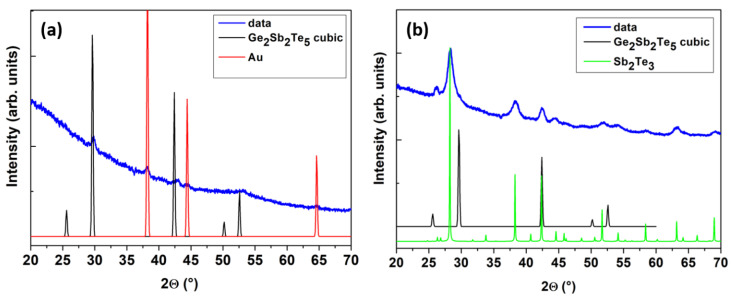
XRD pattern on (**a**) Ge-rich Ge–Sb–Te core NWs, and (**b**) Ge-rich Ge–Sb–Te/Sb_2_Te_3_ core-shell NW, with 50 nm Au NPs on the SiO_2_/Si substrate, respectively.

Figure 2 shows a set of XRD patterns obtained for the Ge-rich Ge–Sb–Te core, and Ge-rich Ge–Sb–Te/Sb_2_Te_3_ core-shell NWs on a SiO_2_/Si substrate. The Ge-rich Ge–Sb–Te core NWs exhibited broad diffraction peaks centered at the 2θ values likely for the face centered cubic (FCC), Ge_2_Sb_2_Te_5_ phases, along with the presence of Au NP diffraction peaks (Figure 2a). No presence of amorphous Ge was detected in the NWs or additional structures, as investigated by SEM. Therefore, the Ge-rich Ge–Sb–Te is crystallized in the cubic structure, with a lattice parameter value similar to that of cubic Ge_2_Sb_2_Te_5_. The additional Ge is likely to be present as an interstitial element in the lattice, causing the spread of the lattice parameter value, which is detected as the enhanced broadness of the diffracted maxima. It should be noted that the cubic GST phase could be simply transformed into the hexagonal structure with a minor atomic rearrangement during the transition process [34]. However, in Figure 2b, for Ge-rich Ge–Sb–Te/Sb_2_Te_3_ core-shell NWs, the GST peak became less intense and narrower, while the diffraction peaks from the Sb_2_Te_3_ phase were clearly obtained. This suggested that no structural disordering occurred after the shell deposition.

**Figure 3:**
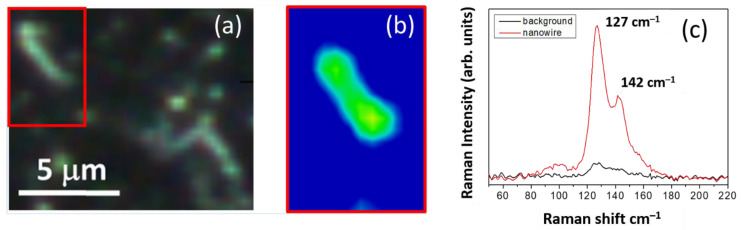
(**a**) Optical image of a Ge-rich Ge–Sb–Te NW identified by Raman microscope on a Si (100) substrate with 50 nm of Au NPs; (**b**) Raman map corresponding to the area indicated by the red rectangle in the optical image; (**c**) Mean Raman spectrum calculated over the wire, green area of the map, compared with that obtained on the background, blue area of the map.

Micro-Raman mapping was carried out on the deposited Ge-rich Ge–Sb–Te core, and Ge-rich Ge–Sb–Te/Sb_2_Te_3_ core-shell NWs to analyze the vibrational modes, obtaining similar spectral results on both SiO_2_/Si and Si (100) substrates. Figure 3a shows the dark field optical image of Ge-rich Ge–Sb–Te NWs identified with a Raman microscope on the Si (100) substrate with Au NPs 50 nm in size. The Raman map corresponding to the area indicated by the red rectangle is presented in Figure 3b and was carried out with a 0.5 µm step size. In Figure 3c, the mean spectrum calculated over the wire (green area of the map) can be clearly distinguished from that obtained on the background (blue area of the map). The NW Raman spectrum mainly showed two peaks at about 127 and 142 cm^−1^, and could be attributed to the FCC-Ge_2_Sb_2_Te_5_ phase [35]. The obtained results are in good agreement with the XRD results, thus the structural and vibrational analysis confirmed the FCC phase of the obtained Ge-rich Ge–Sb–Te NWs.

**Figure 4:**
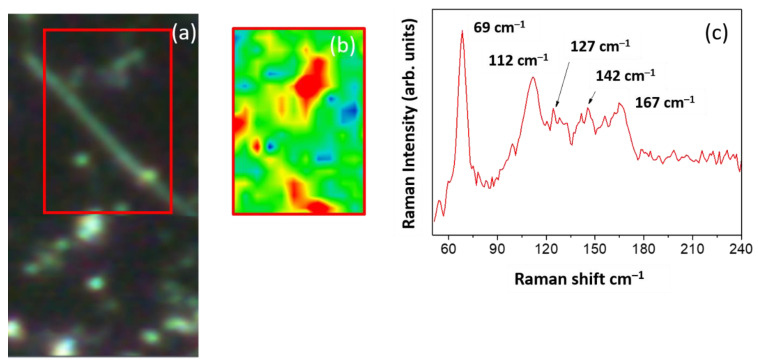
(**a**) Optical image of a Ge-rich Ge–Sb–Te/Sb_2_Te_3_ core-shell NW on a Si (100) substrate with 50 nm of Au NPs identified by the Raman microscope; (**b**) Raman map corresponding to the area indicated by the red rectangle in the optical image; (**c**) Mean Raman spectrum calculated over the wire, red area of the map.

Figure 4 shows the micro Raman image obtained on the Ge-rich Ge–Sb–Te/Sb_2_Te_3_ core-shell NWs on a Si (100) substrate with Au NPs of 50 nm size. A single NW was optically localized and selected by Figure 4a and then spectrally mapped (Figure 4b). Figure 4c reports the mean spectrum calculated over the NW (red area of the map), which shows three dominant peaks at about 69 cm^−1^, 112 cm^−1^, and 167 cm^−1^, associated with the A^1^_1g_ (LO), E^2^_g_(TO), and A^2^_1g_(LO) modes of Sb_2_Te_3_, respectively [36,37,38], in addition to those already identified for Ge-rich Ge–Sb–Te at about 127, and 142 cm^−1^.

**Figure 5:**
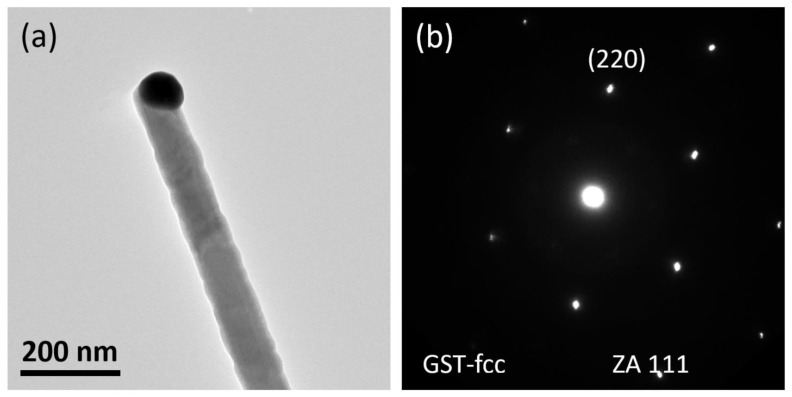
Bright-field TEM micrograph of a Ge-rich Ge–Sb–Te core NW on SiO_2_/Si substrate (**a**) and the relative SAED patterns (**b**), showing a FCC phase.

The HRTEM investigation demonstrated that the obtained NWs have a well-defined core-shell heterostructure, where the core consists of single crystalline Ge-rich Ge–Sb–Te, surrounded by a polycrystalline Sb_2_Te_3_ shell. Figure 5a reports a bright-field TEM micrograph of a Ge-rich Ge–Sb–Te core NW with a size of 90 nm. The image shows that the Ge-rich Ge–Sb–Te NW has a smooth surface with a clearly evident Au catalyst particle at the tip, demonstrating the occurrence of the VLS mechanism during the growth process. The selected area diffraction (SAED) pattern showed regular spot patterns, which further confirmed the perfect single crystalline nature of the NWs. This means that the NWs are made by a single FCC Ge–Sb–Te crystal, as indicated by the respective SAED pattern taken on the trunk (Figure 5b). The EELS analysis (not shown here) confirmed an estimated composition of 35% Ge:10% Sb:55% Te throughout the core NW (see Supplementary Materials, Figure S8), corresponding to the atomic ratio of Ge:Sb:Te = 3:1:5. The obtained composition had a higher Ge concentration with respect to the stoichiometric GST.

**Figure 6:**
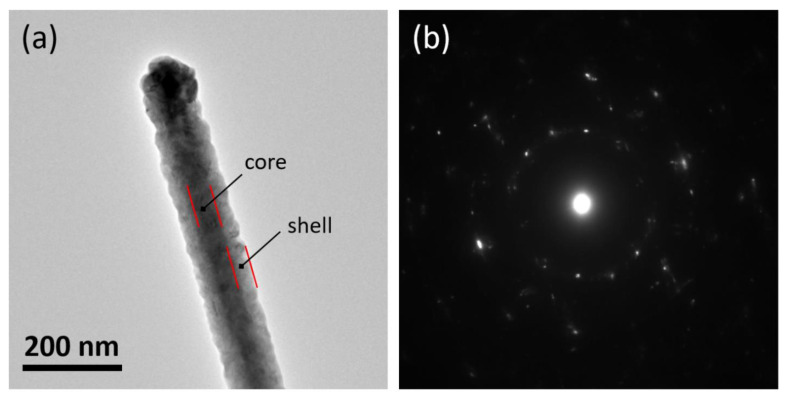
Bright-field TEM micrograph of a Ge-rich Ge–Sb–Te/Sb_2_Te_3_ core-shell NW on the SiO_2_/Si substrate (**a**) and relative SAED patterns (**b**) showing the polycrystalline diffraction pattern from the shell.

Furthermore, the TEM images of Ge-rich Ge–Sb–Te/Sb_2_Te_3_ core-shell NWs indicated a uniform and homogenous conformal overgrowth around the core NWs. In fact, one can clearly observe an interface between the core and the shell region of the NWs, as displayed in Figure 6a, where a bright-field TEM micrograph of a Ge-rich Ge–Sb–Te/Sb_2_Te_3_ core-shell NW with a core size of about 60 nm and a Sb_2_Te_3_ conformal shell about 30 nm thick, is reported. The respective SAED pattern in Figure 6b shows the superposition of a regular diffraction pattern that originates from the monocrystalline core and other dispersed spots set out in the rings around the central spot that originates from the polycrystalline shell. EELS investigation of the polycrystalline shell deposited over the core NW confirmed the existence of only Sb and Te elements, with a stoichiometric ratio of a 2:3, respectively (see Supplementary Materials, Figure S9). In conclusion, the Ge-rich Ge–Sb–Te/Sb_2_Te_3_ core-shell NWs were found without any defects such as stacking faults and dislocations; the crystal structures observed by TEM all over the core and core-shell NW heterostructures were in good agreement with those observed by the XRD analysis.

The above discussed results validate the capability of the presented method in achieving both high quality Ge-rich Ge–Sb–Te core NWs and a uniform Sb_2_Te_3_ shell, conformally deposited at room temperature. This could be achieved while preserving a physical–chemical sharp interface, with minimum elemental inter-diffusion between the two alloys, favored by the room temperature deposition of the Sb_2_Te_3_ shell. These findings also indicate that a reaction between the residual Ge–Sb–Te precursor and the Sb_2_Te_3_ precursor could be avoided and no undesired alloys were formed.

## 4. Conclusions

We reported the optimized MOCVD synthesis of crystalline ternary Ge-rich Ge–Sb–Te (35% Ge, 10% Sb, 55% Te) core nanowires, catalyzed by the VLS mechanism, with a good density. The as-synthesized Ge-rich Ge–Sb–Te nanowires were single crystals in the FCC phase, with an average length up to 1.40 µm, diameter less than 60 nm, and higher in Ge concentration with respect to the stoichiometric Ge_2_Sb_2_Te_5_. The conformal overgrowth of polycrystalline Sb_2_Te_3_ was achieved at room temperature on the previously grown core Ge-rich Ge–Sb–Te nanowires. No structural disordering and core-shell inter-diffusion was evidenced. The obtained chalcogenide core-shell nanowires will be explored for the phase transition behavior, where nanoscale phenomenon and interface effects can be investigated for future embedded PCM cells and automotive applications.

## Data Availability

The data that support the findings of this study are available from the corresponding author upon reasonable request.

## Supplementary Materials

The following are available online at https://www.mdpi.com/article/10.3390/nano11123358/s1, Figure S1: Plan view SEM images on MOCVD growth of Ge–Sb–Te alloy with 10 nm Au NP on SiO_2_/Si substrates, Figure S2: (a) STEM image of as grown Ge–Sb–Te with 10 nm Au NP on SiO_2_/Si substrates at T = 400 °C, P = 50 mbar, t = 120 min, TDMAGe partial pressure = 2.94 × 10^−3^ mbar, SbCl_3_ partial pressure = 2.42 × 10^−3^ mbar, DSMTe partial pressure = 3.52 × 10^−3^ mbar, (b) the composition of the NWs resulted to be mainly that of Ge, as indicated by the EDX spectrum, Figure S3: (a) STEM-EDX images showing the presence of NWs with 10 nm Au NP on SiO_2_/Si substrates at T = 380 °C, P = 300 mbar, t = 180 min, TDMAGe partial pressure = 4.77 × 10^−3^ mbar, SbCl_3_ partial pressure = 1.38 × 10^−3^ mbar, DSMTe partial pressure = 5.37 × 10^−3^ mbar; (b) the composition of the NWs resulted to be mainly that of Sb_2_Te_3_, as indicated by the EDX spectrum, Figure S4: XRD analysis of NWs with 10 nm Au NP on SiO_2_/Si substrates at T = 380 °C, P = 300 mbar, t = 180 min, TDMAGe partial pressure = 4.77 × 10^−3^ mbar, SbCl_3_ partial pressure = 1.38 × 10^−3^ mbar, DSMTe partial pressure = 5.37 × 10^−3^ mbar, Figure S5: Plan view SEM images on MOCVD growth of Ge–Sb–Te nanowires with 50 nm Au NP on Si(100) and SiO_2_/Si substrates at T = 400 °C, P = 50 mbar, t = 60 min, TDMAGe partial pressure = 3.35 × 10^−3^ mbar, (a,b) SbCl_3_ partial pressure = 2.07 × 10^−3^ mbar, DiPTe partial pressure = 7.07 × 10^−3^, (c,d) SbCl_3_ partial pressure = 1.04 × 10^−3^ mbar, DiPTe partial pressure = 7.07 × 10^−3^ mbar, (e,f) SbCl_3_ partial pressure = 3.45 × 10^−4^ mbar, DiPTe partial pressure = 7.07 × 10^−3^ mbar, (g,h) SbCl_3_ partial pressure = 1.73 × 10^−4^ mbar, DiPTe partial pressure = 8.58 × 10^−3^ mbar, (i,j) SbCl_3_ partial pressure = 5.18 × 10^−5^ mbar, DiPTe partial pressure = 8.58 × 10^−3^ mbar, respectively, Figure S6: Plan view SEM images on MOCVD growth of (a–d) Ge-rich Ge–Sb–Te core nanowires, and (e–h) Ge-rich Ge–Sb–Te/Sb_2_Te_3_ core-shell NWs with 10, 20, 30 and 50 nm Au NPs on SiO_2_/Si substrate, respectively, Figure S7: Statistical plot of the average length and diameter distribution of Ge-rich Ge–Sb–Te core nanowires on SiO_2_/Si with 10, 20, 30 and 50 nm Au NPs, Figure S8: STEM image of a Ge-rich Ge–Sb–Te core nanowire portion (a) and its corresponding EELS spectra for Ge L-edge (b) and Sb–Te M-edge (c), Figure S9: STEM image of a Ge-rich Ge–Sb–Te/Sb_2_Te_3_ core-shell nanowire portion (a) and its corresponding EELS spectrum (b), Table S1: Growth parameters corresponding to Figure S1.
