# Supplementary material for: Phase Change Ge-Rich Ge–Sb–Te/Sb2Te3 Core-Shell Nanowires by Metal Organic Chemical Vapor Deposition
Source: Nanomaterials (Basel). 2021 Dec 10;11(12):3358. doi: 10.3390/nano11123358 (PMC8707013; doi:10.3390/nano11123358)
Supplement: Supplementary file 1 [file nanomaterials-11-03358-s001.zip › nanomaterials-1463145-supplementary.pdf]

nanomaterials

MDPI

Supplementary Materials

Phase Change Ge-Rich Ge–Sb–Te/Sb_2Te_3 Core-Shell Nanowires by Metal Organic Chemical Vapor Deposition

Arun Kumar [1], Raimondo Cecchini [2], Claudia Wiemer [1], Valentina Mussi [3], Sara De Simone [3], Raffaella Calarco [3], Mario Scuderi [4], Giuseppe Nicotra [4] and Massimo Longo [3,*]

1 CNR—Institute for Microelectronics and Microsystems, Via C. Olivetti 2, 20864 Agrate Brianza, Italy; arun.kumar@mdm.imm.cnr.it (A.K.); claudia.wiemer@mdm.imm.cnr.it (C.W.)
2 CNR—Institute for Microelectronics and Microsystems, Via Gobetti 101, 40129 Bologna, Italy; cecchini@bo.imm.cnr.it
3 CNR—Institute for Microelectronics and Microsystems, Via del Fosso del Cavaliere 100, 00133 Rome, Italy; valentina.mussi@artov.imm.cnr.it (V.M.); sara.desimone@artov.imm.cnr.it (S.D.S.); raffaella.calarco@artov.imm.cnr.it (R.C.)
4 CNR—Institute for Microelectronics and Microsystems, Strada VIII 5, 95121 Catania, Italy; mario.scuderi@imm.cnr.it (M.S.); giuseppe.nicotra@imm.cnr.it (G.N.)
* Correspondence: massimo.longo@artov.imm.cnr.it

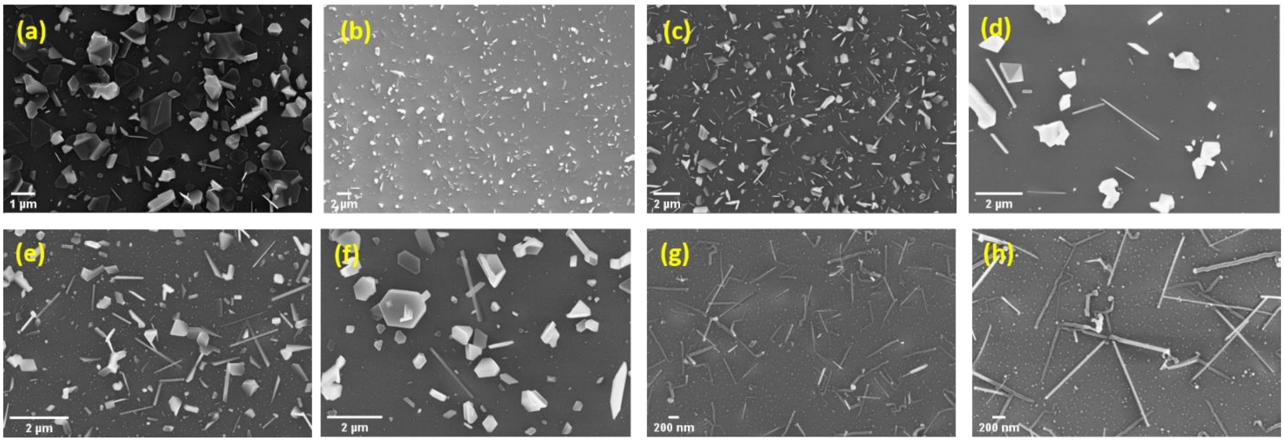

Figure S1. Plan view SEM images on MOCVD growth of Ge–Sb–Te alloy with 10 nm Au NP on SiO_2/Si substrates with the growth parameters mentioned in Table S1, respectively.

Table S1. Growth parameters corresponding to Figure S1.

Growth Parameters / Figure S1	Temperature (°C)	Pressure (mbar)	Time (min)	TDMAGe (Partial Pressure) (mbar)	$SbCl_3$ (Partial Pressure) (mbar)	DSMTe (Partial Pressure) (mbar)
(a)	325	100	120	3.91×10^{-3}	6.04×10^{-3}	8.81×10^{-3}
(b)	370	100	120	7.83×10^{-3}	2.42×10^{-3}	3.52×10^{-3}
(c)	400	50	120	2.94×10^{-3}	2.42×10^{-3}	3.52×10^{-3}
(d)	400	50	120	2.94×10^{-3}	1.21×10^{-3}	8.81×10^{-3}
(e)	375	100	120	1.17×10^{-3}	1.81×10^{-3}	2.64×10^{-3}
(f)	380	100	120	3.91×10^{-3}	1.51×10^{-3}	2.11×10^{-3}
(g)	380	300	120	4.77×10^{-3}	1.38×10^{-3}	5.37×10^{-3}
(h)	380	300	180	4.77×10^{-3}	1.38×10^{-3}	5.37×10^{-3}

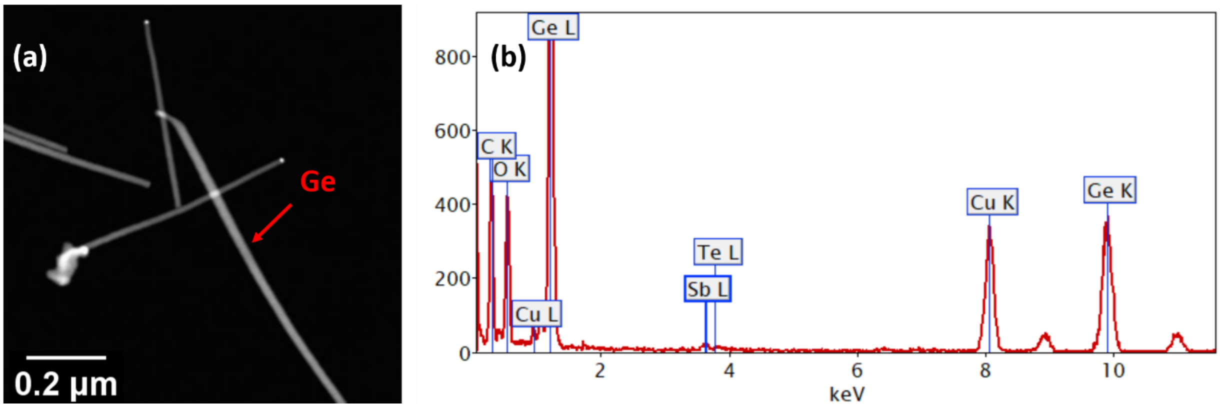

Figure S2. (**a**) STEM image of as grown Ge–Sb–Te with 10 nm Au NP on SiO_2/Si substrates at T = 400 °C, P = 50 mbar, t = 120 min, TDMAGe partial pressure = 2.94 × 10^{-3} mbar, $SbCl_3$ partial pressure = 2.42 × 10^{-3} mbar, DSMTe partial pressure = 3.52 × 10^{-3} mbar, (**b**) the composition of the NWs resulted to be mainly that of Ge, as indicated by the EDX spectrum.

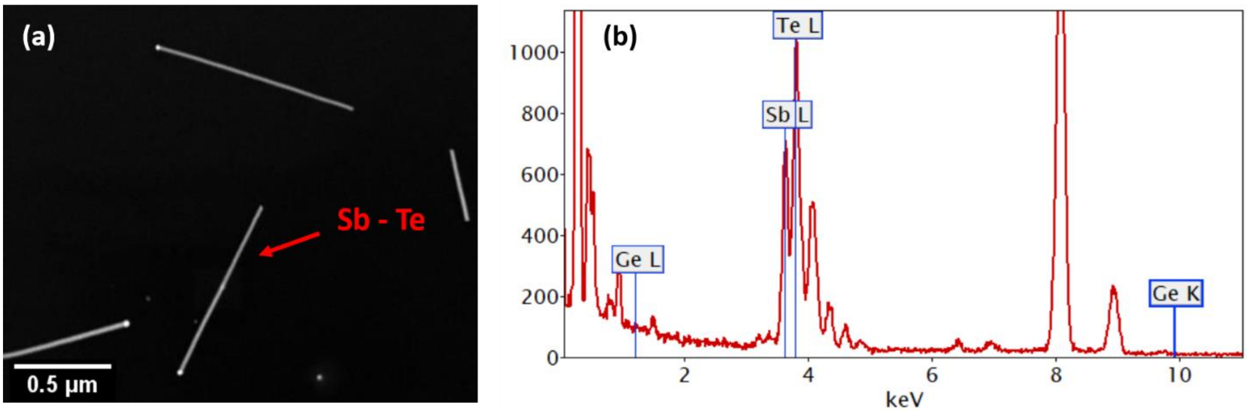

Figure S3. (**a**) STEM-EDX images showing the presence of NWs with 10 nm Au NP on SiO_2/Si substrates at T = 380 °C, P = 300 mbar, t = 180 min, TDMAGe partial pressure = 4.77 × 10^{-3} mbar, $SbCl_3$ partial pressure = 1.38 × 10^{-3} mbar, DSMTe partial pressure = 5.37 × 10^{-3} mbar; (**b**) the composition of the NWs resulted to be mainly that of Sb_2Te_3, as indicated by the EDX spectrum.

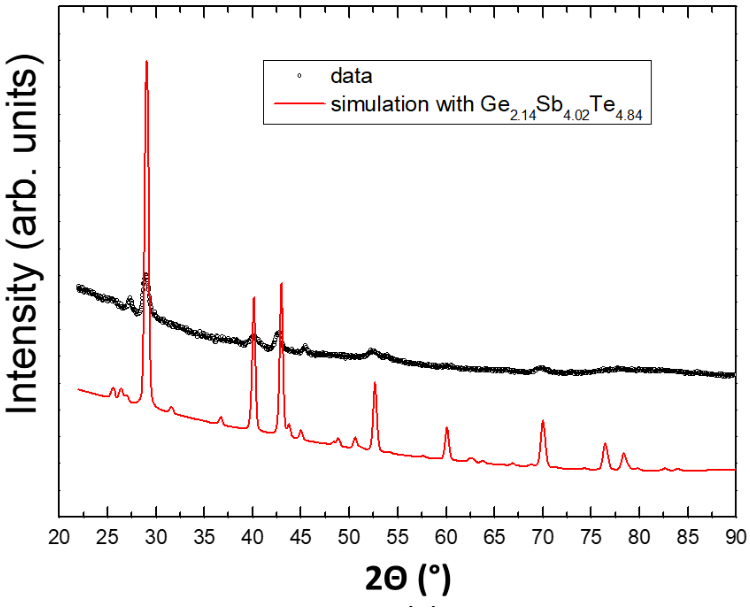

Figure S4. XRD analysis of NWs with 10 nm Au NP on SiO_2/Si substrates at T = 380 °C, P = 300 mbar, t = 180 min, TDMAGe partial pressure = 4.77 × 10^{-3} mbar, $SbCl_3$ partial pressure = 1.38 × 10^{-3} mbar, DSMTe partial pressure = 5.37 × 10^{-3} mbar.

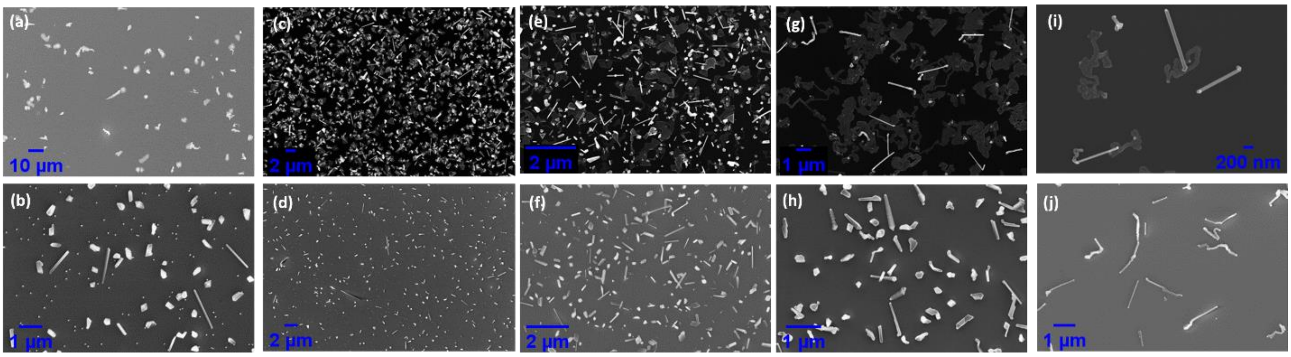

Figure S5. Plan view SEM images on MOCVD growth of Ge–Sb–Te nanowires with 50 nm Au NP on Si(100) and SiO_2/Si substrates at T = 400 °C, P = 50 mbar, t = 60 min, TDMAGe partial pressure = 3.35 × 10^{-3} mbar, (**a**,**b**) $SbCl_3$ partial pressure = 2.07 × 10^{-3} mbar, DiPTe partial pressure = 7.07 × 10^{-3}, (**c**,**d**) $SbCl_3$ partial pressure = 1.04 × 10^{-3} mbar, DiPTe partial pressure = 7.07 × 10^{-3} mbar, (**e**,**f**) $SbCl_3$ partial pressure = 3.45 × 10^{-4} mbar, DiPTe partial pressure = 7.07 × 10^{-3} mbar, (**g**,**h**) SbC_{l3} partial pressure = 1.73 × 10^{-4} mbar, DiPTe partial pressure = 8.58 × 10^{-3} mbar, (**i**,**j**) $SbCl_3$ partial pressure = 5.18 × 10^{-5} mbar, DiPTe partial pressure = 8.58 × 10^{-3} mbar, respectively.

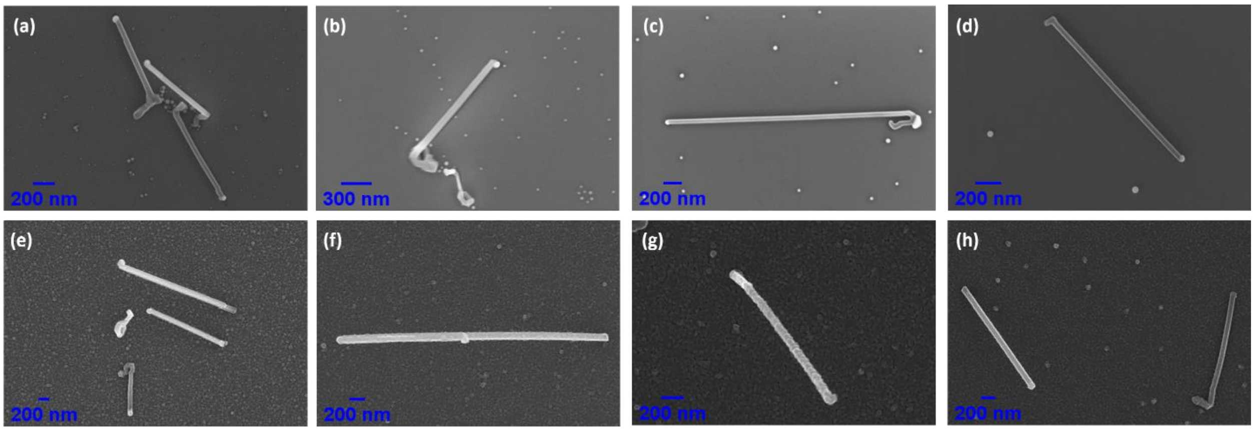

Figure S6. Plan view SEM images on MOCVD growth of (**a**–**d**) Ge-rich Ge–Sb–Te core nanowires, and (**e**–**h**) Ge-rich Ge–Sb–Te/Sb_2Te_3 core-shell NWs with 10, 20, 30 and 50 nm Au NPs on SiO_2/Si substrate, respectively.

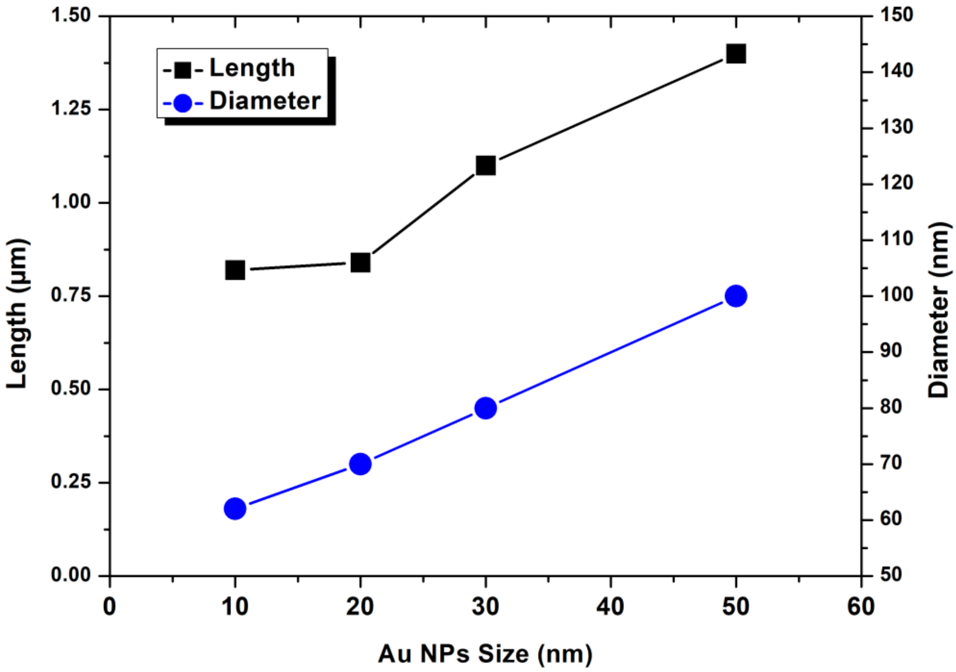

Figure S7. Statistical plot of the average length and diameter distribution of Ge-rich Ge–Sb–Te core nanowires on SiO_2/Si with 10, 20, 30 and 50 nm Au NPs.

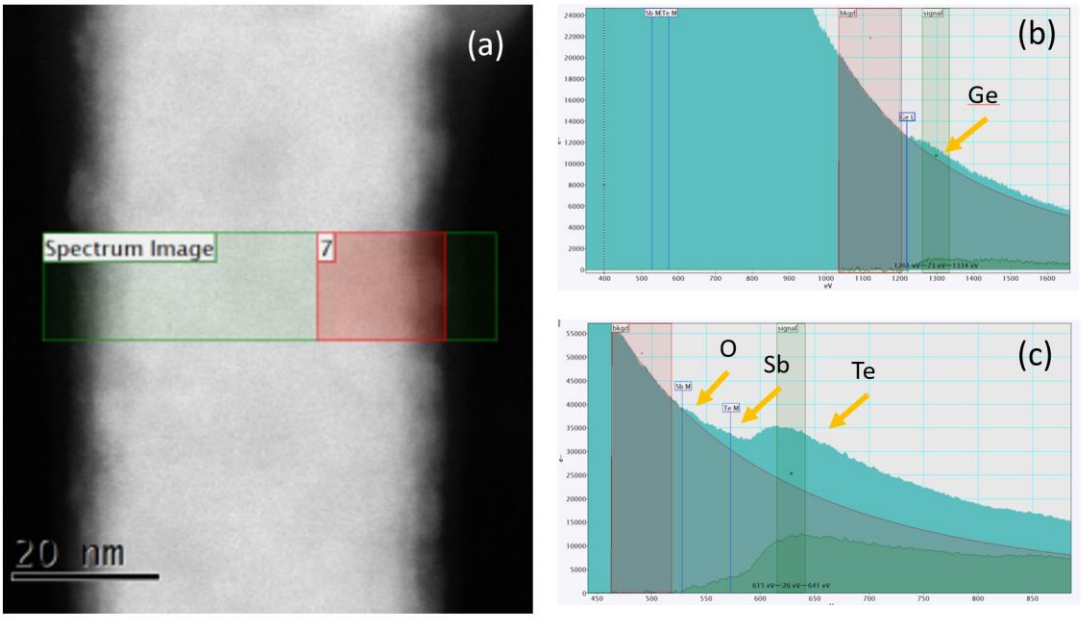

Figure S8. STEM image of a Ge-rich Ge–Sb–Te core nanowire portion (**a**) and its corresponding EELS spectra for Ge L-edge (**b**) and Sb–Te M-edge (**c**).

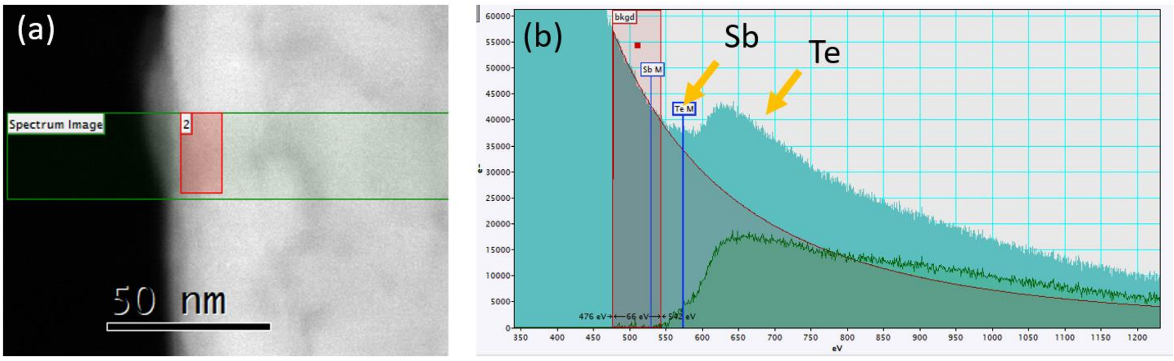

Figure S9. STEM image of a Ge-rich Ge–Sb–Te/Sb_2Te_3 core-shell nanowire portion (**a**) and its corresponding EELS spectrum (**b**).
